# Sustainability in Global Agri-Food Supply Chains: Insights from a Comprehensive Literature Review and the ABCDE Framework

**DOI:** 10.3390/foods13182914

**Published:** 2024-09-14

**Authors:** Gaofeng Wang, Yingying Wang, Shuai Li, Yang Yi, Chenming Li, Changhoon Shin

**Affiliations:** 1 School of Management, Henan University of Technology, Zhengzhou 450001, China; wanggaofeng@haut.edu.cn (G.W.); 2023931111@stu.haut.edu.cn (Y.W.); lishuai@stu.haut.edu.cn (S.L.); 2023931116@stu.haut.edu.cn (Y.Y.); lichenming@stu.haut.edu.cn (C.L.); 2 College of Ocean Science and Engineering, Korea Maritime and Ocean University, Busan 49112, Republic of Korea

**Keywords:** global agri-food supply chains, climate change adaptation, supply chain transparency, innovation-driven development, stakeholder engagement for sustainability, quantitative–qualitative synthesis

## Abstract

The sustainability of global agricultural produce supply chains is crucial for ensuring global food security, fostering environmental protection, and advancing socio-economic development. This study integrates bibliometric analysis, knowledge mapping, and the ABCDE framework to conduct a comprehensive qualitative and quantitative analysis of 742 relevant articles from the Web of Science core database spanning January 2009 to July 2023. Initially, bibliometric analysis and knowledge mapping reveal the annual progression of research on the sustainability of global agricultural produce supply chains, the collaborative networks among research institutions and authors, and the geographic distribution of research activities worldwide, successfully pinpointing the current research focal points. Subsequently, the ABCDE framework, constructed from the quantitative findings, helps us identify and comprehend the antecedents, barriers and challenges, impacts, and driving forces affecting the sustainability of these supply chains. The study identifies globalization and technological advancement as the primary forces shaping the sustainability of agricultural produce supply chains, despite them also posing challenges such as resource constraints and environmental pressures. Moreover, the application of innovative technologies, the optimization of organizational models, and active stakeholder engagement are key to propelling supply chains toward more sustainable development, exerting a profound impact on society, the environment, and the economy. In conclusion, this study suggests future research directions. The integrated methodology presented offers new perspectives and deep insights into the complexities of sustainable global agricultural produce supply chains, demonstrating its potential to foster knowledge innovation and practical applications, providing valuable insights for academic research and policy formulation in this domain.

## 1. Introduction

Global agricultural and food supply chains play a crucial role in food security and the transformation of sustainable food systems [1]. With the deepening of globalization and the continuous growth of the population, ensuring the sustainability of global food supplies has become an issue of concern [2]. According to the statistics of the Food and Agriculture Organization of the United Nations (FAO), the global value of the agricultural and food supply chain has reached several trillion US dollars per annum. To meet the demand for food quantity and diversity in different countries, the global agricultural product supply chain transports food products from areas with low production costs to places in demand, achieving optimized resource allocation and a balanced food supply [3,4]. On the one hand, the establishment of the global agricultural product supply chain has promoted connections between regions with limited agricultural potential and a large population and regions with relative agricultural advantages. This connection has reduced the food supply risk in areas facing food shortages and provided a more diverse and nutritious food selection to meet consumer demand [5]. On the other hand, the global agricultural product supply chain has improved the efficiency of land and water resources through refined production management and advanced logistics technology, reducing resource waste and having a positive impact on the ecological environment [6,7]. Moreover, the rapid emergence of digital technologies such as big data, block chain, and digital twins has provided more opportunities for the development of sustainable global agricultural supply chains [8]. The global agricultural food supply chain (GASC) refers to the entire process that crosses national borders, from the procurement of food production materials to the production, processing, transportation, and sale of agricultural products [9]. It includes the production, food processing, packaging, storage, transportation, and distribution of primary agricultural products, ultimately reaching consumers [10]. The global agricultural and food supply chain has formed an international pattern dominated by the ABCD (ADM, Bunge, Cargill, and Louis-Dreyfus), the four major grain traders. These transnational corporations play a key role in the global grain market through cross-border transactions and have a profound impact on supply chain stability. With the increasing demand for sustainable supply chains, the roles of transnational corporations and smallholder farmers in the supply chain are becoming increasingly prominent [11]. The production methods, livelihoods, and social welfare of smallholder farmers are crucial for the sustainability of the supply chain. In recent years, many countries and international organizations have actively developed and implemented a series of policies and measures to enhance the sustainability of the agricultural and food supply chain. For example, the European Union has issued the “Farm to Fork” strategy, and the United States Department of Agriculture also launched multiple initiatives aimed at improving supply chain sustainability.

However, this supply chain also faces numerous challenges, including the impact of climate change, natural disasters, policy changes, and market fluctuations. These challenges not only threaten global food security, but also affect the livelihoods of farmers and economic stability. Firstly, the energy consumption and carbon emissions from long-distance transportation and logistics activities place a certain burden on the environment [12,13,14]. Secondly, the complexity of tariffs, insurance, export and import procedures, as well as non-tariff measures and standards in different countries, increase trade costs and affect the efficient operation of the supply chain [15]. In addition, the global agricultural product supply chain faces risks such as natural disasters, political conflicts, trade policy uncertainty, and speculative behavior, which have potential impacts on food security and agricultural stability [16,17]. Therefore, building a secure, circular, high-quality, and efficient global agricultural product supply chain is of great importance for ensuring global food security. This is not only related to the economic development and social stability of various countries, but also directly affects the balance of supply and demand, price stability, and the diversity and nutritional richness of food in the global agricultural market.

To address these challenges, the academic community needs to strengthen its research in this field, promoting the practice and innovation of global sustainable agriculture. Literature reviews are of significant value and necessity for advancing research progress, supporting policy development, and promoting practical application [18]. By summarizing and integrating previous research findings, literature reviews can form a systematic knowledge framework, revealing the key issues and challenges of the sustainability of the global agricultural product supply chain, and providing a basis for research and policy development [19,20]. However, the existing research largely focuses on specific segments or regions of the supply chain, and lacks systematic research on the entire global supply chain. Furthermore, there is no unified theoretical framework or practical guide on how to effectively integrate sustainable development goals (SDGs) with the sustainable building of agricultural food supply chains. In terms of research content, scholars mainly focus on the sustainable agricultural supply chain [21] and the global sustainable supply chain [22,23], but there is a lack of research on the sustainability of the global agricultural product supply chain. In terms of research methods, quantitative or qualitative single analysis methods are mainly used [24,25,26], and quantitative and qualitative methods have not been combined in the research. In terms of research frameworks, traditional literature reviews often just summarize and induct research [27,28,29,30], and have not yet established a holistic, comprehensive research framework, which would be conducive to better understanding the problems and exploring solutions. This study innovatively integrates quantitative data visualization from CiteSpace with the qualitative analysis of the ABCDE framework (antecedents, barriers, challenges, drivers, and effects), aiming to construct a comprehensive research framework that deepens the understanding of the sustainability issues in global agricultural supply chains. This approach not only reveals key influencing factors, but also captures emerging and peripheral research trends, providing new perspectives and guidance for both academia and practice. With this in mind, this study comprehensively analyzes the research status of the global agri-food supply chain’s sustainability based on relevant literature indexed in the Web of Science core database from January 2009 to July 2023. The following research questions are proposed:

RQ1: What is the current overview of the annual publication output, core authors, author cooperation networks, publishing institutions and countries, and high-frequency keywords in global sustainable agricultural supply chains?

RQ2: What are the antecedents, barriers and challenges, drivers, and effects of the global sustainable agricultural supply chain according to the ABCDE framework?

RQ3: What are the future research directions in sustainable agricultural supply chains?

The remaining content of this study is arranged as follows. Firstly, Section 2 introduces the research methods, data sources, and search formulas. Section 3 conducts a visual knowledge mapping analysis of global agricultural sustainability supply chain-related literature from aspects such as the temporal characteristics of publication volume, core authors and author collaboration networks, countries and institutions, and domain keywords. Section 4 integrates the ABCDE framework for analysis, presenting a comprehensive research perspective on the global agricultural sustainability supply chain from four aspects: causes, obstacles and challenges, driving factors, and impacts. It also provides an integrated review and summary of the framework as a whole and the interconnections between its various components. Section 5 outlines the significance of this study and its research hotspots, proposing future research directions for the sustainable development of global agricultural products. Finally, the conclusions and a future outlook are described.

## 2. Research Methods and Data Sources

### 2.1. Research Methods

Bibliometrics are an important discipline that applies mathematical and statistical methods to quantitatively analyze literature [31]. CiteSpace software (6.2.R3) is a powerful tool that can represent and visualize the development of a discipline through maps, but research has found that its application in the study of the sustainability of the global agricultural product supply chain still needs to be deepened. Therefore, this study uses CiteSpace for bibliometric analysis, examining aspects such as publication time, authors, institutions, countries, and high-frequency keywords.

When using CiteSpace software to create a network diagram of author collaborations, the size of the nodes reflects the number of articles published by each author, while the thickness of the connecting lines represents the strength of the collaboration between researchers. The overall network density illustrates the general level of collaboration among authors. Node centrality refers to the degree of connection of a node with other nodes. If the centrality of a node is ≥0.1, it indicates good centrality, signifying close connections with other nodes. Keywords are highly condensed on the topic of a paper, and they also represent the core ideas of a paper. An in-depth analysis of keywords in relevant literature provides a comprehensive understanding of the current research status and hotspots in the field [32]. When using CiteSpace software, selecting the node type “Keyword” generates a co-occurrence graph of keywords. Using this graph, a cluster analysis of keywords will reveal the strong associations among them. Additionally, analyzing the timeline of the keyword co-occurrence graph presents the evolution of the research hotspots. This method provides an insightful approach to understanding the development of the field, offering a comprehensive and intuitive perspective on the evolution and dynamics of research. This authors of this paper conducted a cluster analysis of thematic keywords in the research on global sustainable agricultural supply chains through keyword clustering and the LLR (log-likelihood ratio) algorithm. The cluster analysis was conducted using two criteria: the modularity value Q and the average silhouette value S. The graph was considered significant when Q was greater than 0.3, and the clustering was considered reasonable when S was greater than 0.5. Moreover, the closer S is to 1, the more reasonable the clustering effect [33]. This study used the keyword burst method to analyze the selected literature and explore the trends in the research. The assumption was that the burst strength of a specific keyword in a specific field is unusually high. In this case, it indicates a significant increase in research on that topic within the time frame, reflecting the research hotspots in global sustainable agricultural supply chains in different years [34]. This presented the changes in research hotspots and provided strong support for qualitative analysis of research topics.

However, a single quantitative research method may obscure the deep-level insights and theoretical frameworks brought by qualitative research, which are crucial for understanding the social, cultural, and economic complexities within supply chains. The limitations of quantitative data, such as bias and the difficulty of obtaining data, may lead to a one-sided understanding of issues, and the diversity and interdisciplinary nature of supply chains mean that quantitative methods may simplify the complexity and dynamics of the problems. At the same time, quantitative research often overlooks historical and contextual backgrounds, which should not be neglected in the study of agricultural supply chains. Therefore, to gain a comprehensive and in-depth understanding, literature reviews should integrate qualitative and quantitative research methods to reveal the diversity and multi-dimensional characteristics of sustainable agricultural supply chains.

In this study, we adopted an innovative fusion approach that combines the qualitative ABCDE framework with the quantitative CiteSpace tool to achieve a comprehensive analysis of the sustainability issues in the global agricultural product supply chain. The integration of CiteSpace and the ABCDE framework offered complementary research methodology advantages. Through the qualitative ABCDE framework, we delved into the complexity of the supply chain, identifying key factors affecting its sustainability, including potential challenges and drivers. Meanwhile, the quantitative analysis of CiteSpace provided a data-driven perspective [35], revealing research trends and patterns through visualization, offering an empirical basis for qualitative analysis. This integration allowed us to examine issues from multiple dimensions, ensuring the depth and breadth of the research. It not only enriched our understanding of the sustainability issues in the global agricultural product supply chain, but also provided new perspectives and insights for future research, offering substantial support for the construction of a more sustainable food supply chain system.

### 2.2. Data Sources

To ensure that the literature sources used in this study were of high quality and relevance, the Web of Science (WOS) Core Collection database was selected. WOS is renowned for its collection of high-quality journal literature from both domestic and international sources [36], and it is more suitable for conducting large-scale and highly reliable bibliometric analyses. When setting the conditions for literature retrieval, full consideration was given to the unity of the source scope, consistency of measurement, the scale of quantity, balance of quality, and the authority of impact [37].

For the theme of this study, “Sustainability in Global Agri-Food Supply Chains: Insights from A Comprehensive Literature Review and the ABCDE Framework”, repeated discussions and reflection resulted in four levels of keyword groups ultimately being determined for the literature retrieval. These four levels are “global”, “agricultural products”, “supply chain”, and “sustainability”. Through repeated experiments in WOS, these keywords were input into the retrieval system and four keyword groups were identified to ensure the retrieval of literature resources that were comprehensive and relevant to the research topic (the search used only studies published in English).

**Figure 1:**
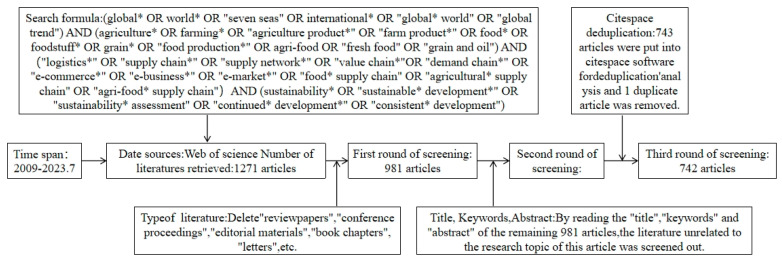
Sample literature screening flow chart. Chart source: self-made by the author.

The search formula used in the WOS database, which integrates four levels of keywords through Boolean logical operators, is as follows: (global* OR world* OR international* OR “global* world” OR “global trend”) AND (agriculture* OR farming* OR “agriculture product*” OR “farm product*” OR food* OR foodstuff* OR grain* OR “food production*” OR agri-food OR “fresh food” OR “grain and oil”) AND (“logistics*” OR “supply chain*” OR “supply network*” OR “value chain*” OR “demand chain*” OR “ecommerce*” OR “e-business*” OR “e-market*” OR “food* supply chain” OR “agricultural* supply chain” OR “agri-food* supply chain”) AND (sustainability* OR “sustainable* development*” OR “sustainability* assessment” OR “continued* development*” OR “consistent* development”). Using precise screening, 742 documents that were highly relevant to the research topic of this paper were obtained and exported in plain text format. For details on the specific screening process and criteria, see Figure 1.

## 3. Visualization and Analysis of Publication Trends

### 3.1. Analysis of Publication Volume Trends over Time

**Figure 2:**
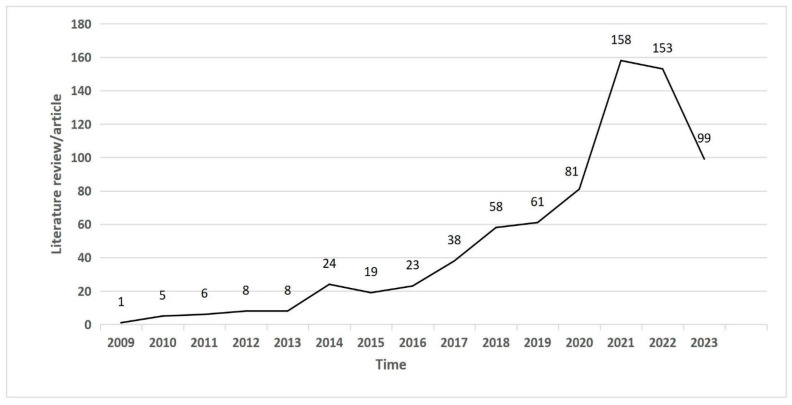
Trends in the number of articles issued, from January 2009 to July 2023. Data source: based on the WOS core database; chart source: self-made by the author.

The visualization of annual publication volume is a key tool for studying developments and trend changes in a particular field [38]. The research on the sustainable development of agricultural supply chains over the past 15 years has shown an overall trend of fluctuating growth, which can be seen in Figure 2. This period can be divided into three stages: the first stage is the stable period (2009–2013), during which there were relatively few research results, with the first study published in Bioresource Technology in 2009. This involved the development of computer models to determine changes in each link of ethanol processing and optimize the interaction of new technologies by adjusting the logistics costs of product delivery, thus increasing the sustainability of the entire industry [39]. The second stage was the rising period (2014–2018), during which 162 articles were published, accounting for 22% of the total. The number of studies on the sustainable development of agricultural supply chains gradually increased during this period. The third stage was the rapid growth period (from 2019 to July 2023), during which 552 articles were published, accounting for 74% of the total. In 2021, the publication volume reached a peak (158 articles), an increase of 95% from 2020. This was mainly due to the expansion of emerging technologies such as digitalization, the importance of basic agricultural supply chains in various countries’ policies, and the increased recognition of ecological sustainability development [40,41]. At the same time, it also showed that during the lockdown period in 2020–2021 a large number of scholars focused on writing, completing, and submitting their papers, which we can see from the number of publications significantly reducing after 2022, even though it is still higher than in 2020. As the article data end in July 2023, it can be foreseen that the sustainable development of global agricultural supply chains will receive more attention in the future, and the publication volume is expected to continue to grow. 

### 3.2. Analysis of Authors’ Publication Patterns

**Figure 3:**
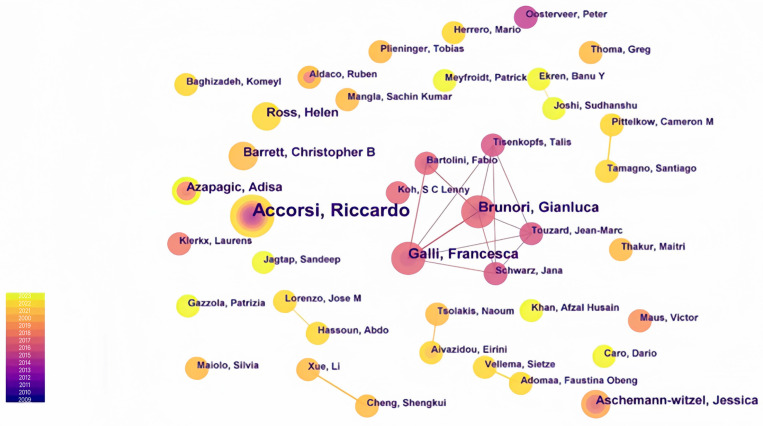
Mapping of author collaboration networks. Chart source: self-made by the author.

Analyzing the collaborative network of authors helps reveal the core group of authors and their collaborative relationships in global agricultural products’ sustainable supply chains [42]. This study used CiteSpace software to draw a network diagram of author collaborations (see Figure 3). This indicates that in regard to the “Global Agricultural Product Sustainable Supply Chains”, collaboration among researchers is relatively limited and there are many independent authors. This result provides valuable insights into the collaboration patterns among researchers in this field.

(1) $$M\approx 0.749\sqrt{Nma}x$$

According to Formula (1), where *M* represents the minimum number of publications by core authors, *Nmax* represents the number of publications by the most prolific author in the field [43].

As shown in Table 1, in this study, the scholar with the highest number of publications was Accorsi, Riccardo, who has published a total of 7 papers; therefore, *Nmax* = 7. When substituting these values into Formula (1) and rounding *M*, we obtained 1.982. According to Price’s law, authors who have published more than 2 papers can be considered core authors. Therefore, Accorsi, Riccardo (7 papers); Brunori, Gianluca (4 papers); Galli, Francesca (4 papers); Barrett, Christopher B (3 papers); Ross, Helen (3 papers), and others were identified as core researchers in this field. Statistical analysis revealed that the total number of publications by core authors in the research sample is 149 papers, accounting for 20.1% but not exceeding 50%, indicating that a well-defined core author group has not yet emerged in this field.

The author with the most publications primarily focused on the quality assessment of food supply chains and proposed an evaluation framework and related data analysis structures. Other authors, either collaboratively or individually, concentrated on research aspects such as the functional analysis of global land and water resources in supply chains [44], the development vision and potential opportunities and challenges of future sustainable food supply chains [45], achieving sustainable development goals in the global agricultural product industry [46,47], tools to reduce the complexity of food system research [48], economic market analysis of small-scale agriculture [49], and life cycle assessment studies of various production systems [50]. When conducting quantitative analysis of the global agri-food supply chain (GASC) using CiteSpace, each segment focuses on the following research areas. In the field of production, research focuses on the intelligence and automation of agricultural production, analyzing research on the Internet of things (IoT) [51], precision irrigation systems, and agricultural robots in terms of improving agricultural production efficiency and reducing resource consumption. In the processing segment, emphasis was placed on innovative technologies in the food processing process, such as the application of nanotechnology in food processing, microbial control during food processing [52], and the high-value utilization of by-products from food processing. In the packaging segment, research was conducted on sustainability in the packaging process, analyzing the ecological design of packaging materials, consumer acceptance of environmentally friendly packaging, and how packaging innovation affects consumer purchasing decisions [53]. In the storage segment, the exploration of energy-saving technologies in the storage process was emphasized, such as the use of advanced insulating materials, intelligent temperature control systems, and the impact of the storage process on food quality and safety [54]. In the transportation segment, the focus was on the optimization of the transportation process, analyzing optimization algorithms for transportation networks, real-time monitoring technologies during transportation, and the impact of the transportation process on the carbon footprint of the food supply chain [55]. In the distribution segment, research was conducted on market dynamics in the distribution process, analyzing the diversity of distribution channels, the role of e-commerce in food distribution [56], and the influence of distribution strategies on consumer behavior. The research conducted by authors in this field demonstrates the characteristics of diversity, comprehensiveness, and interdisciplinary complexity.

### 3.3. Institutions and Countries Analysis

**Figure 4:**
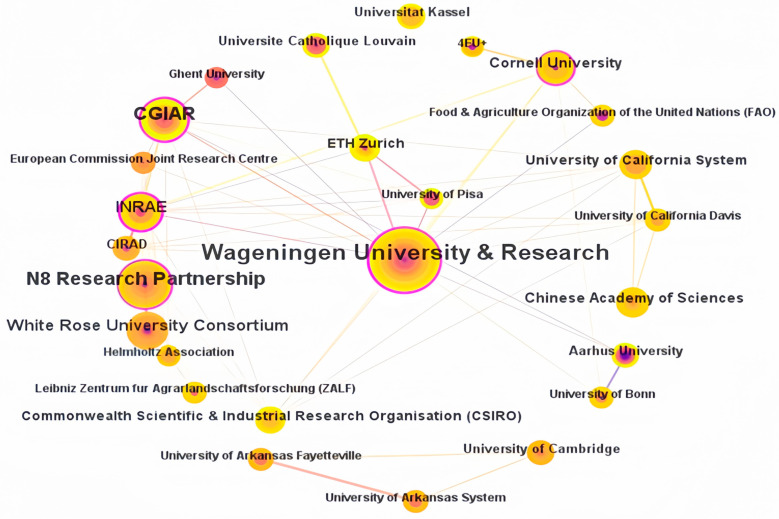
School organizations and institutions mapping chart. Chart source: self-made by the author.

The number of publications reflects the academic level and influence of research institutions in a particular field, as it is generally believed that the number of publications positively correlates with academic level and influence [57]. As shown in Figure 4, the nodes in the graph represent authoritative school organizations and institutions in the “global agri-food supply chains” field, and the nodes’ sizes represent the institutions’ publication volume. The institutional collaboration network graph analysis shows 307 publishing organizations in the sample, with 655 connections between the organizations. The network density of institutional collaboration is 0.0139, indicating that there are relatively many organizations researching cross-border agricultural product supply chains, but collaboration between research institutions is limited.

**Figure 5:**
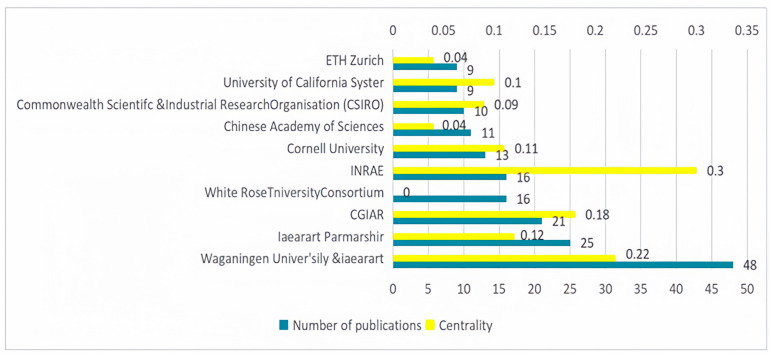
Statistics of publication numbers and centrality of the top ten organizations. Data source: based on the WOS core database; Chart source: self-made by the author.

Figure 5 shows that INRAE had the highest centrality at 0.3, followed by Wageningen University & Research at 0.22, and CGIAR at 0.18. A few research institutions had a centrality of less than 0.1 in global sustainable agri-food supply chains. Moreover, high centrality does not necessarily correlate with high productivity, as seen with INRAE. Therefore, institutions with solid centrality should intensify their research efforts and increase their publication output to leverage a radiating effect within the field. 

Wageningen University & Research (48 papers) had the highest number of publications. Other organizations with a significant number of publications included the N8 Research Partnership (25 papers), CGIAR (Consultative Group on International Agricultural Research) (21 papers), White Rose University Consortium (16 papers), and INRAE (National Research Institute for Agriculture, Food and Environment) (16 papers). In terms of the organizations’ overall contributions, the top three institutions making the most significant contributions to global agri-food supply chains are Wageningen University & Research, CGIAR, and the University of California System. Regarding the types of research organizations, those involved in global agri-food supply chain research are primarily concentrated in universities and research organizations. Wageningen University & Research, one of the world’s top universities, leads significantly in terms of its publication volume in this field.

**Figure 6:**
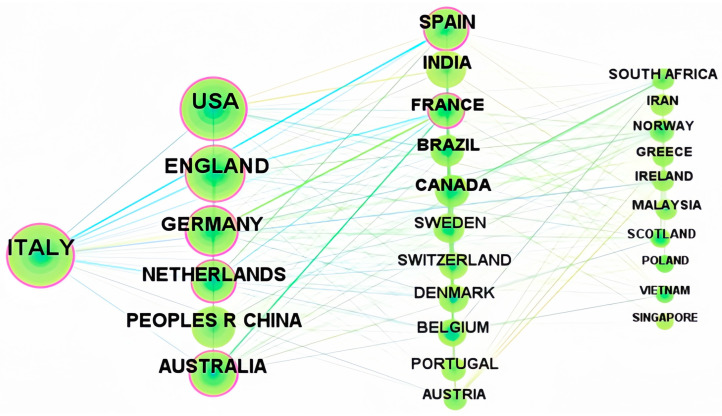
Mapping of cooperation among issuing countries. Chart source: self-made by the author.

Hotspot issues often emerged and were closely correlated with a country’s social and economic environment. Using the CiteSpace tool, with the “node types” set to “country” and other settings unchanged, a global collaborative network graph of countries contributing to research on sustainable agri-food supply chains was generated, as shown in Figure 6. The 742 sample documents were distributed across 104 countries and regions, with 12 countries having produced over 30 papers.

**Figure 7:**
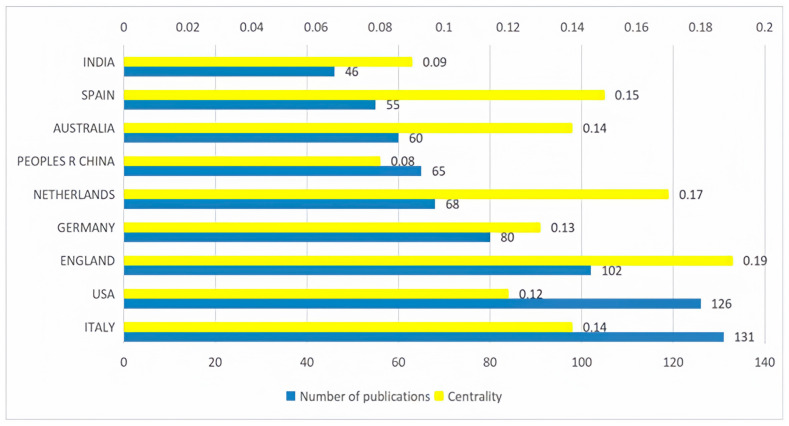
Number of publications and centrality statistics for the top ten countries. Data source: based on the WOS core database. Chart source: self-made by the author.

The network diagram in Figure 6 illustrates the collaboration in publishing among countries. The network contains 780 connections, and the network density of the country collaboration network reached 0.1456. Notably, regarding publication hotspots, most of the top ten countries have centrality indicators exceeding 0.1, as detailed in Figure 7. This indicates a relatively high network density, suggesting that research activities in this field are relatively concentrated in these countries, and a close collaborative relationship exists among them. This close collaboration provides strong support for addressing the challenges faced by global sustainable agri-food supply chains.

As shown in Figure 7, Italy leads with 131 papers, constituting 17.7% of the total output. The United States of America follows closely with 126 papers, representing 17.0% of the total, and England ranks third with 102 papers. Additionally, Germany (80 papers), the Netherlands (68 papers), China (65 papers), and Australia (60 papers) are also prolific in the global research on sustainable agri-food supply chains. 

Italy’s leadership in the number of published papers indicates that the country is very active in the research of sustainable agricultural food supply chains. The United States and the United Kingdom account for a certain proportion of the total number of papers, demonstrating the research strength and contributions of these two countries in this field. In addition, Germany, the Netherlands, China, and Australia also have a considerable number of published papers, indicating that these countries also play an important role in the global research on sustainable agricultural food supply chains. These data reflect the increasing global attention on sustainable agricultural practices and innovation in food supply chains, and also highlight the potential research strengths and leadership of these countries in promoting research in this field.

### 3.4. Global Sustainable Agri-Food Supply Chain Hotspot Analysis

#### 3.4.1. High-Frequency Keyword Co-Occurrence Analysis

**Figure 8:**
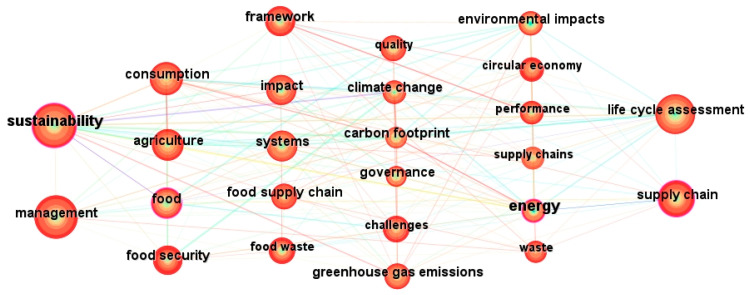
Keyword co-occurrence map. Chart source: self-made by the author.

Keyword co-occurrence analysis helps us better grasp the current research hotspots and predict future research directions. In the graph, the size of the nodes directly reflects the frequency of keyword co-occurrence. As shown in Figure 8, the results show a total of 60 high-frequency keywords related to the research content of the selected articles; “sustainability” (119 times) occupies an absolute core position in the graph, followed by “management” (114 times), “life cycle assessment” (101 times), and “supply chain” (75 times) (numbers in parentheses indicate the frequency of keyword occurrence, and “year” is the year in which the keyword first appeared in the document sample). From this analysis, it is evident that research hotspots in the field of global sustainable agri-food supply chains mainly focus on areas such as “life cycle assessment”, “food security”, “food waste”, and “climate change”. For detailed frequency and year information, please refer to Table 2. This comprehensive analysis provides a deep understanding of the research trends and critical areas, offering guidance for future academic discussions and practices.

When considering the strength of the correlation between keyword frequency and relevance to the research direction, the division of high and low importance keywords is based on the formula [58].

(2) $$T=(-1+\sqrt{1+8\times I_{1}})/2$$

In the formula, *T* represents the minimum frequency of occurrence for high frequency keywords, and *I_1_* is the number of keywords that occur only once. When *T* = 9, keywords appearing nine times or more are considered high-frequency. 

#### 3.4.2. Keyword Cluster Analysis

**Figure 9:**
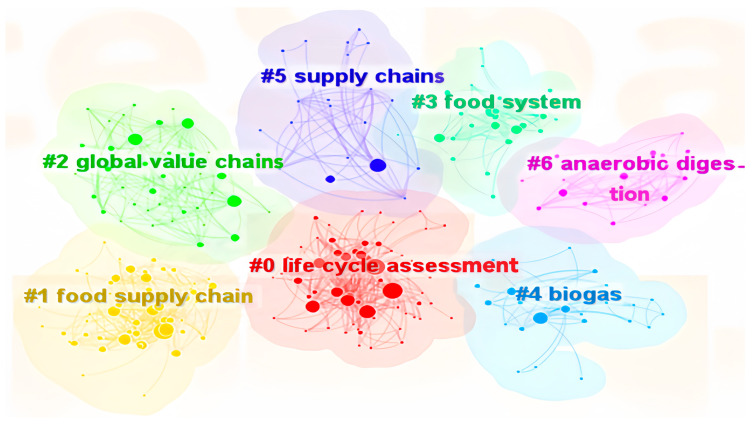
Knowledge graph of keyword clustering in the global sustainable supply chain of agricultural products. Chart source: self-made by the author.

Through keyword clustering and the LLR algorithm (log-likelihood ratio), seven cluster labels were identified: “life cycle assessment”, “food supply chains”, “global value chains”, “food system”, “biogas”, and “anaerobic digestion” (Figure 9). Based on the demonstration of the clustering graph and the display of keywords related to clustering labels, it was found that research clusters were more focused on life cycle analysis, ecosystems, food systems, supply chains, and food supply chains, as well as global value chains. The Q value is 0.3464 and the S value is 0.6663, indicating that the clustering graph is both significant and reasonable.

**Figure 10:**
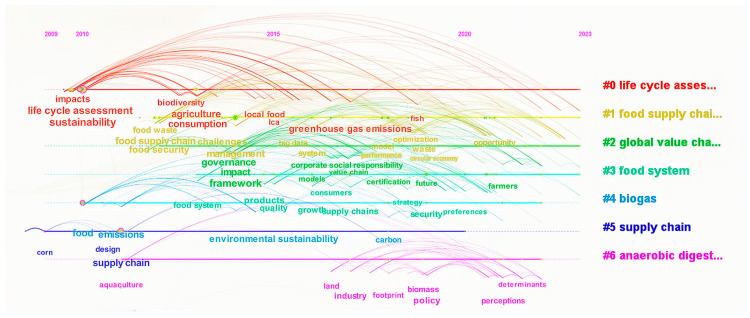
Global sustainable supply chain research timeline. Data source: based on the WOS core database. Chart source: self-made by the author.

A global timeline chart of sustainable agricultural supply chains was created through further analysis of keywords, as shown in Figure 10. Combining the analysis of the timeline chart, it was observed that, except for “supply chain”, which is relatively isolated, the other keywords related to global sustainable agricultural supply chains are closely interconnected. It can be seen that clusters such as “life cycle assessment” (2010), “food supply chains” (2012), “global value chains” (2012), “food system” (2013), and others have been consistently associated with research in the field of agricultural supply chains. These topics have long been focal points for scholars studying agricultural supply chains. Although the topics “biogas” (2010–2022), “supply chain” (2009–2020), and “anaerobic digestion” (2011–2022) emerged early and have been studied for a relatively long time, their continuity has not been constant. 

The analysis indicates that while the keyword “supply chain” stands relatively independently, others such as “life cycle assessment”, “food supply chain”, “global value chains”, and “food systems” have formed tight clusters, demonstrating the sustained research interests and interconnectivity of these topics between 2010 and 2013. In addition, themes that emerged early like “biogas”, “supply chain”, and “anaerobic digestion”, though fluctuating in their persistence in the research, have laid the foundation for subsequent studies, potentially indicating the initial growth points and early exploratory stages of their research interest. The early and prolonged presence of these topics underscores their significance in shaping the knowledge system of sustainable agricultural supply chains. Overall, these findings provide an in-depth understanding of the evolution of global sustainable agricultural supply chain research and offer clear directions and key areas for future research, especially for further exploration of methodologies and practical applications.

#### 3.4.3. Keyword Burst Analysis

**Figure 11:**
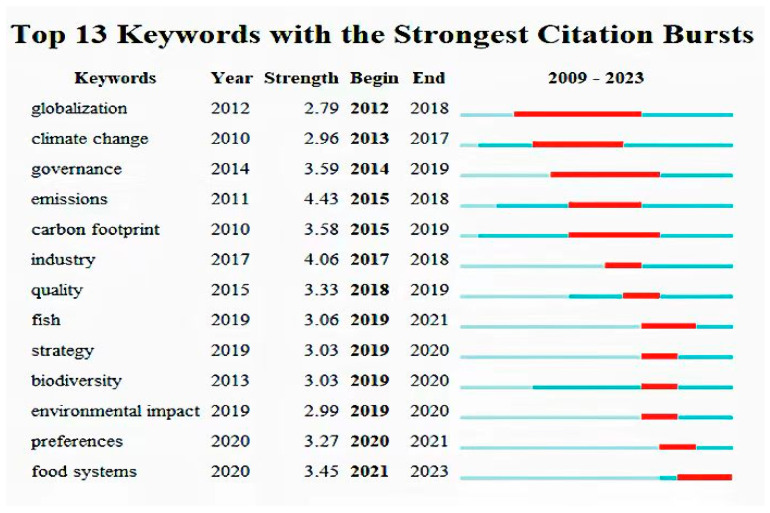
Atlas of emerging words in the global sustainable supply chain of agricultural products. Data source: based on the WOS core database. Chart source: self-made by the author.

Utilizing CiteSpace software for burst analysis of keywords in the global sustainable agricultural supply chain field allowed for an in-depth understanding of the forefront research issues in this domain. Bursting words are terms that experience a sudden and significant frequency increase within a specific period [59]. This study delved into 13 bursting keywords, with detailed information available in Figure 11. Here, “keywords” represents the keywords, “strength” represents the burst strength, “begin” represents the starting year, “end” represents the ending year, and the red lines represent the burst periods of the keywords.

Among these 13 bursting keywords, the keyword with the highest burst strength was “emissions”, with a strength of 4.43 and a duration of 3 years (2015–2018). In contrast, the keyword with the lowest burst strength was “globalization”, with a strength of 2.79 and a duration of 6 years (2012–2018). Among these 13 keywords, 12 have concluded their bursting periods, and the only keyword that has not yet terminated its burst is “food systems”, indicating that it is currently a frontier research hotspot in this field. This burst analysis revealed the evolution of research keywords, with early bursting keywords including “globalization” and “climate change”, mid-term bursting keywords encompassing “governance”, “emissions”, “carbon footprint”, etc., and late-term bursting keywords concentrating on “biodiversity”, “environmental impact”, and “food systems”, highlighting research on ecological and environmental protection and food system optimization. What is particularly noteworthy is that “environmental impact” and “food systems” may become future research hotspots.

## 4. Optimizing Sustainability in the Global Agri-Food Supply Chain through the ABCDE Framework

This study comprehensively analyzed research in the field of global agricultural product supply chain sustainability through the combination of bibliometrics and knowledge mapping methods. The keyword co-occurrence diagram and institution collaboration network diagram present the research status and key hotspots. After a visual analysis of 742 samples, we carefully read the contents of the literature. We found that previous research in this field can be divided into four aspects: ABCDE (antecedents, barriers and challenges, driving factors, and effects), so we proposed the ABCDE framework and explained these findings in detail in the fourth part. The ABCDE framework reveals the origin, problems faced, driving forces, implementation effects, and potential impacts in the field. The antecedent analysis reveals the origin and motivations of sustainable agricultural supply chains. The obstacles and challenges section identifies potential problems that may arise in achieving sustainability goals. The driver analysis focuses on the key factors that drive development. Finally, the impact section comprehensively evaluates the implementation effects and potential long-term impacts of sustainable supply chains.

### 4.1. Antecedents

The antecedents refer to the factors that affect the sustainability of the global agricultural supply chain, such as globalization, market growth, changes in consumer demand, climate change, the development of policies and regulations, and technological advancements.

Due to the rapid industrialization of agriculture and increasing attention on food quality and safety, the concepts of sustainability and supply chain transparency have become crucial for agriculture and the agricultural food sector [60]. At present, although global food production has significantly increased, factors such as climate change, energy security, and international regional conflicts pose multiple threats [61]. The uneven distribution of food and the changes in regional dietary structures, coupled with economic development and changes in consumer demand, jointly promote the urgent need for the global agricultural food supply chain to transform into a diversified supply system [62].

Human factors: Global population growth and urbanization have increased demand for agricultural products. Scholars have studied social capital, including partnerships, social networks, and trust, as well as the role of sustainable entrepreneurship in enhancing global agricultural supply chain sustainability [63]. Additionally, they have focused on how consumers’ demand for and attitudes toward sustainable agricultural products impact the supply chain, as well as the influence of rural economic development and social change on agricultural supply chains [64]. In summary, these factors collectively influence the operation and development of sustainable food supply chains.

Internal factors: Researchers have assessed the impact of sustainable development goals (SDGs) on various stages of the global agricultural supply chain. Attention has been given to applying sustainability assessment tools to food supply chains, introducing different quantitative assessment methods and indicators. Furthermore, some scholars have addressed the sustainable procurement challenges and practices in the global food industry, emphasizing the impact of procurement on the overall sustainability of the supply chain [65]. Simultaneously, research on agricultural production technologies, such as precision agriculture and organic farming [66], has been conducted to understand their impact on the supply chain. These research directions contribute to a more comprehensive understanding of sustainable agriculture and food supply chains at various levels, facilitating the achievement of sustainable development goals.

External factors: From the perspective of climate change, researchers have focused on its impact on the supply chain [67], including agricultural adaptation measures and strategies to mitigate climate change, as well as the impact of increased consumption of natural resources (land, water resources, and energy) on the global sustainability of agricultural supply chains. Emphasis has been placed on the importance of collaboration and the involvement of multiple stakeholders in promoting the sustainability of agri-food supply chains [68]. Additionally, it highlights the importance of considering ecological, biodiversity, social fairness, and economic factors in sustainability [69]. Studies have also pointed out the crucial role of government policies and support in developing sustainable agricultural supply chains. In contrast, the complexity and uncertainty of different countries’ policies, regulations, and trade agreements can also impact the supply chain [70]. In the production segment, the focus is primarily on research into the sustainability of agricultural practices, including the application of precision agriculture techniques [71], the construction of ecological agricultural systems, and the optimization of crop management. The studies emphasize the adoption of water-saving irrigation technologies, organic farming, and measures for the protection of biodiversity, aiming to enhance the ecological efficiency of agricultural production and reduce its negative environmental impacts. Together, these research directions reveal the significant role of external factors in shaping the sustainability of agricultural supply chains.

### 4.2. Barriers and Challenges

Barriers and challenges include resource constraints, environmental pressures, market demand instability, technological backwardness, insufficient policies and regulations, as well as the complexity brought by globalization. These factors may hinder the efficient operation and long-term sustainability of the supply chain.

Climate change, natural disasters, and the trend towards financialization and energy conversion of agricultural products have collectively exacerbated the volatility of agricultural product prices and increased the vulnerability of the agricultural food supply chain [72]. This vulnerability manifests as the risk of fragility and disconnection of the supply chain, especially with the impact of geopolitical tensions and extreme natural disasters, which pose challenges to the highly concentrated export supply of global agricultural trade.

Scarcity of resources and environmental pressure: Resource constraints and environmental issues, such as water pollution, greenhouse gas emissions [73], environmental and external uncertainties, soil degradation, and rising energy costs have adverse effects on the supply chain. Unstable weather events triggered by climate change threaten agricultural products’ production and supply chains [74]. Large-scale agricultural production has led to ecosystem destruction and biodiversity loss [75], negatively impacting the long-term sustainability of agriculture. Additionally, food waste and losses exist at various stages of the agricultural supply chain, from cultivation to transportation, processing, and retail, placing additional pressure on resources and the environment.

Market demand: International markets are witnessing an increased demand for sustainable agricultural products, but consumer behavior may be influenced by price, brand, and convenience, posing a challenge [76]. Research has been dedicated to enhancing consumer awareness of sustainable products to motivate changes in their purchasing behavior. Market systems need help with issues, with cost-efficiency being a significant obstacle, especially in agricultural pricing and distribution, where price fluctuations impact the stability of the supply chain [77]. Therefore, research has focused on how market system reforms can reduce costs and decrease market uncertainty.

Technological backwardness: Some regions still employ traditional agricultural practices and technologies, limiting the development of sustainable supply chains. Research in the storage segment has focused on enhancing the energy efficiency of storage facilities and adopting advanced food preservation technologies. This includes improvements to cold chain technologies [78] to reduce spoilage during storage and ensure the freshness and quality of food throughout the entire supply chain. Research in the transportation segment focuses on optimizing logistics networks and transportation modes to reduce greenhouse gas emissions from the supply chain [79]. It explores the use of multimodal transport systems, electric and alternative energy vehicles, as well as the potential of real-time transportation management systems to improve transportation efficiency and reduce costs. Research has been committed to integrating sustainable practices into technological innovations. The agricultural supply chain involves multiple stages [80] and coordinating and managing technology is crucial for achieving a sustainable supply chain. Researchers have explored methods for cultivating agricultural talent to promote rural sustainable development [81]. Many farmers need advanced agricultural technology and knowledge of sustainable agricultural practices [82], posing constraints on their productivity and sustainability.

Policy and regulatory insufficiency: While policies and regulations support the development of sustainable agricultural supply chains, there are areas for improvement in implementation and supervision. Lack of adequate policy support and legal frameworks leads to unfavorable agricultural practices [83], affecting the establishment and operation of sustainable supply chains. Agricultural labor often results in unfair working conditions, low wages, and a lack of social security policies, negatively impacting the social sustainability of the agricultural supply chain. Information asymmetry and opacity resulting from globalization and international trade make government decision-making difficult and thus increase risks. Insufficiencies in policies, regulations, and management pose challenges to various aspects of the agricultural supply chain, including environmental and social aspects.

### 4.3. Driving Factors

The driving factors refer to the key elements that propel the supply chain towards a more sustainable development, such as innovative technologies (e.g., blockchain, the Internet of things), organizational models, quality management, risk mitigation strategies, and the involvement and cooperation of stakeholders.

Organizational patterns: Scholars have emphasized the importance of integrating sustainability principles into supply chain organizational patterns. They have studied the establishment of resilient supply chain organizational patterns, focusing on sustainable products and supply chain transparency to adapt to changing market demands [84]. Additionally, attention has been given to embedding risk management strategies in organizational patterns [85] to enhance the supply chain’s resilience. Vertical coordination has been proposed as part of organizational pattern innovation. The application of transaction cost theory and principal agent theory has been used to analyze agricultural supply chain organizational patterns, emphasizing the impact of transaction costs on the choice of supply chain channel patterns. These research directions collectively highlight the crucial role of supply chain organizational patterns in achieving sustainability and improving operational efficiency.

Quality assurance: Research has focused on enhancing food safety and quality in the agricultural supply chain [86], including studies on food detection technologies, risk assessment, hygiene standards, and more. Researchers have strived to improve traceability and transparency in the supply chain through technological means such as blockchain, the Internet of things (IoT), and other technologies [87]. They have focused on raising food safety in the agricultural supply chain, covering environmental and social sustainable development goals, greenhouse gas emissions [88], food additives, etc. Simultaneously, the research has addressed establishing and improving quality standards and certification systems for agricultural products to ensure product quality. In the processing segment, research was concentrated on enhancing energy efficiency and reducing the generation of waste during processing. Innovations in food processing technology were explored, such as non-thermal preservation techniques [89], effective utilization of by-products, and the reduction of carbon footprints and water resource consumption in food processing. These research directions collectively emphasize the critical role of quality assurance in ensuring food safety and quality in the agricultural supply chain.

Risk mitigation: Researchers have focused on various risks in the agricultural supply chain [90], including natural disasters, supply interruptions, and market demand fluctuations. They have explored methods of mitigating risk transmission and proposed corresponding risk management strategies [91]. In addition, they have studied approaches to mitigating financial risks in the supply chain using financial tools and insurance mechanisms [92]. Attention has been given to slowing down risk transmission through supply chain collaboration and information sharing, proposing corresponding risk management strategies [93]. Researchers have also examined the formulation of reasonable insurance policies to support agricultural production and mitigate risks in the agricultural supply chain. They have also studied how to leverage information technologies, such as the Internet of things and big data, to achieve information sharing in the agricultural supply chain [94]. In the distribution segment, research was focused on enhancing the transparency and responsiveness of the supply chain, as well as reducing uncertainty and risk within the food supply chain. The application of blockchain technology in ensuring the traceability and quality safety of food products was discussed [95], along with the effectiveness of demand-driven distribution strategies to reduce food waste and improve consumer satisfaction. These research directions highlight the critical role of risk mitigation in agricultural supply chain management, covering multiple aspects to enhance the stability and sustainability of the supply chain. 

### 4.4. Effects

Effects refers to the profound influence of supply chain sustainability on human society, the environment, and the economy. This includes improving food safety and nutritional value, reducing the environmental footprint, enhancing social well-being, and strengthening economic sustainability.

Effect on people: Some researchers have analyzed the post-green revolution food system, exploring how to improve the global agricultural supply chain to address the triple burden of malnutrition, including hunger, malnutrition, and being overweight/obese [96]. Sustainable supply chains can ensure a stable, high-quality food supply [97]. Researchers have pointed out that sustainable supply chains help enhance food safety and nutritional value, emphasizing the implementation of agricultural product quality regulations and food safety standards and meeting consumer demand for healthy food [98]. These studies highlight the crucial role of sustainable supply chains in improving the global food system and addressing nutritional issues.

Effect on the environment: Considering environmental and social characteristics can enhance the effectiveness of food quality policies. Some scholars have focused on evaluating the environmental sustainability of agricultural supply chains, emphasizing the importance of sustainable agri-food supply chain management in achieving global food safety and environmental protection [99]. They have used methods such as life cycle assessment to analyze the environmental impact of various stages of the agricultural supply chain, including aspects such as carbon footprint, energy use, and waste management [100]. Sustainable supply chains help reduce the negative impact of agricultural activities on the environment [101], improve agricultural production methods, and reduce the use of chemical pesticides and fertilizers, contributing to ecological protection. In the packaging segment, research has emphasized the development of environmentally friendly packaging materials such as biodegradable plastics and bio-based materials. Additionally, the impact of packaging design on reducing food waste has been studied [102], along with the application of smart packaging technologies to extend the shelf life of food products and enhance food safety. These studies highlight the importance of environmental sustainability in agricultural supply chain management to promote food safety and ecological conservation. 

Effect on society: Some scholars have discussed social sustainability issues in agricultural supply chains, including considerations of agricultural labor conditions, community engagement, and land rights [103]. The responsibilities of all parties in the agricultural supply chain, public awareness, and demand drive the development of food supply chains towards sustainable goals. Sustainable supply chains help increase farmers’ income and quality of life, reduce poverty, and emphasize the participation and benefits of rural communities [104]. These studies highlight the critical role of social sustainability in agricultural supply chain management to ensure the rights and well-being of agricultural laborers and their communities.

Effect on the economy: Regarding economic sustainability, scholars have delved into issues related to the agricultural food supply chain [105]. They have analyzed costs, efficiency, and profits at different stages and examined the impact of improving the sustainability of the entire supply chain on maintaining economic sustainability [106]. This would require reshaping the supply chain dynamics and improving stakeholder relationships, as well as enhancing cooperation and transparency. Interdepartmental and international cooperation is crucial for sustainable food supply chain development. Researchers have also studied how the development of sustainable supply chains contributes to promoting rural economic diversification and the rise of rural cooperatives [107], with a focus on enhancing the sustainability of rural communities through rural economic development. These studies highlight the critical role of economic sustainability in agricultural supply chain management to ensure the profitability of the supply chain and promote the diversification and sustainable development of the rural economy.

### 4.5. Summary of the ABCDE Framework for Global Agri-Food Sustainable Supply Chains 

**Figure 12:**
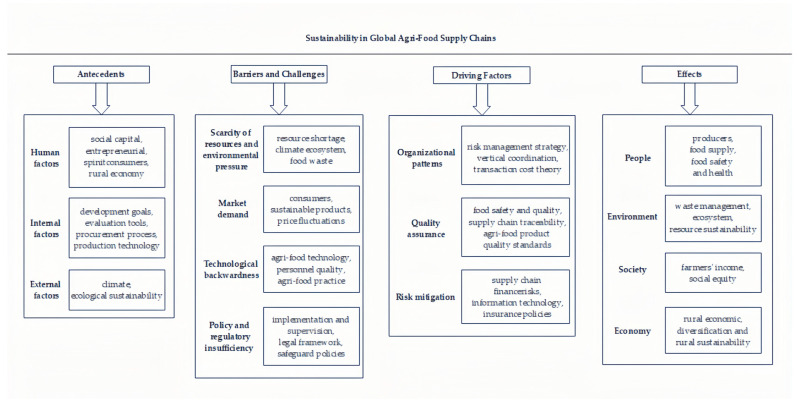
The ABCDE framework for sustainable global agri-food supply chains. Chart source: self-made by the author.

The analysis of global agricultural sustainable supply chain development using the ABCDE framework revealed several core findings (refer to Figure 12). Firstly, the research emphasizes the diversity and interrelatedness of sustainability issues in global agricultural supply chains. Neglecting any category or factor may lead to an incomplete understanding of the problems, highlighting the urgency of a comprehensive consideration of the issues. Considering the importance of each factor in the ABCDE framework contributes to a more comprehensive resolution of global agricultural supply chain sustainability issues. Secondly, achieving sustainable development in global agricultural supply chains requires meeting multiple prerequisites, emphasizing the urgency of addressing foundational issues. This study identified various driving factors propelling the sustainable development of agricultural supply chains, covering economic, social, technological, and other aspects of motivation. Overcoming various barriers and challenges makes it difficult to achieve sustainability, highlighting the complexity and the requirement of comprehensive strategies. Thirdly, sustainable development has multifaceted impacts on people, society, the environment, and the economy, and calls for a comprehensive consideration of social equity, ecological environment, and economic benefits in assessments. While some studies mentioned obstacles and challenges, the existing literature maintains a positive outlook on sustainable global agricultural supply chains. It is crucial to comprehensively address the sustainability issues in global agricultural supply chains by considering all factors in the ABCDE framework to avoid an incomplete understanding of the problems. Additionally, the research found that some factors appeared in more than one category, such as traceability, food safety, and quality, which can simultaneously be considered driving factors and influencing outcomes. This emphasizes the close association between internal driving factors, social equity, and economic development. In the relationship between prerequisites and barriers and challenges, a lack of prerequisites may become a barrier or challenge to achieving sustainable development, including challenges like technological advancement vs. insufficient skills, or reluctance to provide data vs. information sharing. This interrelationship needs to be comprehensively considered; some factors are being viewed as prerequisites, while others are seen as obstacles and challenges. Finally, an unclear and tense situation exists when exploring the relationship between driving factors and barriers and challenges. Resolving this relationship requires a balance between perceived importance and perceived feasibility. If the driving factors are deemed necessary, efforts will be made to overcome barriers and challenges. However, if these obstacles are considered insurmountable, they may hinder the push for sustainable development. Balancing this tension is crucial for the sustainable development of global agri-food supply chains.

## 5. Discussion

### 5.1. Significance of the Research

This study comprehensively applied CiteSpace visualization analysis and the ABCDE framework to thoroughly examine research on the sustainability of global agricultural food supply chains from January 2009 to July 2023. Our analysis revealed the research dynamics in the field, the contributions of core scholars, and key research hotspots, providing the academic community with valuable information resources and trend analysis. Compared with the existing literature, our study results offer new perspectives. In particular, we found that although modern algorithms and technologies have made progress in optimizing the sustainability of supply chains [108], issues such as unclear research objectives [109,110], deviation from research directions [111,112,113], and weak communication among researchers [114] still exist. 

The development of a globally sustainable agricultural food supply chain is a key focus for the future of agriculture [115]. Better coordination between national institutions and individuals in this field, as well as control over the trends and pain points of global agricultural supply chains, is crucial for producing high-quality research outcomes. Research on sustainable agricultural supply chains helps us ensure global food security [116], understand how to increase agricultural productivity while reducing resource consumption, improve the quality of agricultural products, and ensure that people have access to adequate and diverse nutrition. Agricultural supply chains involve a wide range of socio-economic issues, and research on global sustainable supply chains is closely related to the United Nations sustainable development goals (SDGS), especially those concerning zero hunger, clean water, sustainable cities and communities, and poverty eradication [117]. This is essential for ensuring global food security, protecting the environment, maintaining social equity, and achieving sustainable development goals [118,119]. Furthermore, the findings of this study can provide insights for policy-makers, educational reformers, and industry practitioners to help them improve the decision-making process and practice methods. By presenting an interdisciplinary perspective and complementing or challenging existing theories, this research provides new research questions and theoretical frameworks for academia, while emphasizing the innovation of research and enlightenment in academia, with both theoretical and practical significance.

(1) Theoretical significance.

① Visualization of supply chains. Through the analysis of a number of published articles, we can gain an understanding of the development history and current research hotspots in this field, and thereby predict future research directions. The analysis of authors and institutions can help identify influential researchers and leading institutions in this field, promoting the optimal allocation of academic resources. Moreover, the analysis of countries and keywords revealed the research priorities and differences of various countries and regions in the resilience and sustainability of agricultural product supply chains, providing a basis for international academic exchanges and collaborations;

② Establishing a theoretical framework for sustainability supply chains. Through dynamic analysis of keywords, we can track and evaluate the evolution of theoretical development, providing support for the construction of a systematic theoretical framework for supply chain sustainability. Additionally, this process involved identifying and integrating research achievements from various disciplinary fields, such as economics, management science, and ecology, to promote interdisciplinary research;

(2) Practical significance.

① Enhancing the efficiency of supply chain management. By analyzing the changes in the number of published articles, companies and policy-makers can understand the trends of market and technological development, to improve the foresight and adaptability of their supply chain management. The analysis of publishing institutions and countries was helpful to find best practices in supply chain management, and promote the exchange and learning of experiences among countries;

② Promoting international trade and cooperation. Understanding and analyzing the research status and policy orientations of different countries in the agricultural supply chain is conducive to finding opportunities and ways for international cooperation. Promoting the formulation and implementation of international standards can enhance the cross-border compatibility and the interoperability of supply chain;

③ Strengthening supply chain risk management. Through the analysis of key words, we found the key factors affecting the sustainability of the supply chain, thus helping companies identify and manage potential risks. Promoting supply chain transparency can enhance consumer confidence and improve market competitiveness;

④ Supporting sustainable development strategies. Identifying and promoting models and practices can achieve sustainable development while enhancing supply chain resilience, and provide decision support for policy-makers to formulate policies and measures that promote the sustainability of agri-food supply chain. The research results not only provide theoretical support and practical guidance for the sustainable development of the agricultural supply chain, but also contribute to the goal of establishing a safe, stable, and sustainable global food system in the United Nations 2030 Agenda for Sustainable Development.

### 5.2. Research Hotspots

Based on the analysis results from CiteSpace and the ABCDE framework, considering the currently significant challenges and opportunities in the field of global agricultural sustainable supply chains and the changing trends in global agri-food systems, the following research hotspots are proposed:

① Environmental dimension: The impact of climate change on agriculture and the food system has become a focus of global attention [120,121]. Extreme climate events and changes in rainfall patterns all have an impact on the sustainability of agricultural supply chains, requiring in-depth research on coping strategies. In the promotion of life cycle analysis, environmental sustainability considerations gradually attract people’s attention and life cycle analysis provides a comprehensive understanding of the environmental impact of agricultural supply chain [122,123]. 

The concept of circular economy: The rise of the circular economy concept promotes agriculture from a linear production mode to a circular economy [124,125] so as to reduce resource waste and improve the efficiency of resource utilization;

② Technology dimension: The application of information technology, big data, artificial intelligence, and other digital technologies in agriculture is gradually increasing [126], which puts forward new challenges and opportunities to improve the efficiency and sustainability of the agri-food supply chain. Network security problems have been highlighted. With the wide application of information technology, network security issues have become increasingly important [127]. The agri-food product supply chain involves a large amount of data exchange and information sharing, requiring a better network security guarantee;

③ Social and economic dimension: The agriculture and food industries are closely related to society and the economy [128]. Therefore, studying the impact of agricultural supply chains on employment, social equity, and rural development becomes crucial. With increased consumer demand for sustainable products, attention to product traceability and sustainability increases, which introduces new requirements for the management and standard setting of agricultural supply chains. With regard to policy and regulation promotion, countries’ focus on sustainable development has prompted governments to introduce relevant policies and regulations, which have a direct impact on the sustainability of the agricultural supply chain;

④ Supply chain dimension: Global events (such as pandemics, climate change impacts, etc.) pose new challenges to global supply chains [129], so improving the sustainability of supply chains becomes crucial. The UN sustainable development goals provide a common direction for the world, and the sustainability research of agricultural supply chains needs to be coordinated with these goals.

### 5.3. Research Agenda

By integrating CiteSpace and other visualization techniques into research on the sustainability of the global agricultural product supply chain, proposed future research directions include: 

① Enhancing supply chain transparency and visualization by developing and applying new technological tools such as blockchain and increasing the number of programs invested in practical environments to ensure visibility into the use of pesticides and fertilizers by various global agricultural supply chain entities [130], thus improving the transparency of the agricultural supply chain and enhancing the sustainability of the entire system;

② Strengthening international cooperation using linkage programs to expand the linkage framework and enhance interactive trade across scales and regions [131]. Also, encouraging and strengthening international cooperation in agricultural supply chain management and sustainable development research to jointly address global challenges such as climate change and pandemics;

③ Researching how to enhance the sustainability of agricultural product supply chains through comprehensive risk management strategies, and introducing ASC risk management quantitative models and other multi-faceted evaluations, as well as assessing and managing risk factors such as natural disasters, market fluctuations, and policy changes [132];

④ Supporting smallholder farmers and local agriculture through research on how to improve the position of small farmers in the supply chain and promote the sustainable development of local agriculture through technological and policy support. For example, building aggregation and scaling policies or digital technology sinking areas to promote the coordinated industrialization of small-scale agricultural markets [133]. Through in-depth exploration of these research directions, we can promote the sustainability of agricultural supply chains and contribute to building a more stable and efficient global agricultural supply chain system.

① Antecedents: The lack of efficiency and transparency in agricultural supply chains has led to compromised food safety and consumer confidence [134]. The growth of globalization and market demand, coupled with consumers’ higher expectations for food safety, has driven the need for digital transformation of supply chains. However, weak infrastructure [135], particularly in transportation, storage, and processing, has become a bottleneck for sustainable development. Therefore, research should focus on leveraging information technology to enhance supply chain transparency [136] and reduce waste, adapting to globalization and changes in market demand, addressing the complexity of cross-border supply chains [137], improving supply chain management through the development of advanced software and data analysis tools, and investing in infrastructure to enhance operational efficiency and sustainability;

② Barriers and challenges: The global agricultural product supply chain faces multiple challenges in the pursuit of sustainability. The digital divide [138], organizational structural issues, unclear investment returns, and globalization-related challenges such as disasters and pandemics all threaten supply chain transparency and efficiency. To overcome these challenges, research should focus on technological innovations to enhance supply chain sustainability [139] and security, safeguarding food security and international cooperation to stabilize global supply chains [140], developing cross-border data security standards [141] and promoting data flow, exploring optimized organizational structures and collaboration models, and assessing the economic benefits of sustainable development strategies [142] to encourage investment;

③ Drivers: Global agricultural product supply chains have numerous driving factors in the pursuit of sustainable development. Existing research frameworks provide a foundation for exploration, but the specific application of technologies such as blockchain, Internet of things, big data, and artificial intelligence in agricultural supply chains needs to be further deepened. Future research directions should focus on the application of digital technologies, exploring how blockchain [143], the Internet of things, and other technologies can enhance supply chain efficiency and transparency, studying the impact of cutting-edge technologies such as the metaverse and digital twins on agricultural product traceability and market regulation [144], utilizing big data and artificial intelligence to predict market dynamics and optimize production decisions, and specifically analyzing the practical application and details of technological implementation in agricultural supply chains [145];

④ Effects: Research in the field of sustainable agricultural supply chains has a profound impact on production and daily life. While the ABCDE framework provides an overview of research, further exploration is needed into how to achieve ESG guidelines, dual carbon strategies, and green development. Future research directions should focus on implementing ESG guidelines and building sustainable agricultural production and supply chains that align with ESG principles [146], analyzing the implementation path of dual carbon strategies in the agricultural sector and the impact of green development on nutritional balance [147], exploring supply chain network optimization and benefit sharing mechanisms [148] such as integrated production, sales, and cross-border resource allocation strategies, studying how supply chain transparency enhances consumer confidence [134] and increases brand value, analyzing how organizational configuration optimization improves supply chain synergies, reduces costs, and enhances adaptability, and exploring how supply chains can support the United Nations 2030 Agenda goals, such as poverty elimination [149], health improvement, and sustainable development.

These research directions can help achieve a more sustainable, safer, greener, and more circular agricultural supply chain to meet the growing global food demand and reduce the negative impact on natural resources. They reflect the continuous evolution of the field of sustainable agricultural supply chains to adapt to new challenges and opportunities, as well as the societal goals for sustainable development and concerns for environmental protection. Research in these areas will help meet future needs and challenges.

## 6. Conclusions and Future Outlook

### 6.1. Conclusions

Based on a comprehensive analysis of 742 papers retrieved from the Web of Science core database, this study employed quantitative and qualitative methods, including CiteSpace for bibliometrics and knowledge graph analysis and the ABCDE framework for content analysis.

CiteSpace analysis revealed an overall upward trend in research activity in the field from 2009 to 2019, with a significant surge in publications from 2020 to July 2023. The peak occurred in 2021 with 158 papers, followed by a slight decline in 2022. Noteworthy institutions such as Wageningen University & Research, the N8 Research Partnership, and CGIAR demonstrated prominent performance despite the relatively limited collaboration between institutions. However, there was a concentration of research activity at the national level, with close collaborative relationships. Italy, the United States, and the United Kingdom emerged as significant contributors, with Italy leading in the number of publications. Regarding core authors, Riccardo Accorsi stood out as having the highest number of publications, contributing seven relevant articles. The author’s collaboration network exhibited diversity and was interdisciplinary with loosely connected relationships. Keyword analysis revealed that “supply chain” appeared to be relatively isolated, while other keywords exhibited tight interconnections. Specific keywords, such as “biogas”, “supply chain”m and “anaerobic digestion” played initial roles in the early stages of agricultural product supply chain research. Keywords like “life cycle assessment”, “food supply chains”, “global value chains”, and “food system” remained consistently associated with the field throughout its development.

Through a visual analysis of 742 articles in related fields, we gained a deep understanding of the current research status of the sustainable agri-food supply chain. However, although these analyses provided us with valuable information, they do not fully reveal the specific directions of future research. To address this gap, we propose the ABCDE framework, which is designed to provide a solid theoretical basis and guidance for future research. Based on the ABCDE framework, the content analysis provided a comprehensive perspective on the sustainable development of the global agricultural product supply chain. The analysis of antecedents revealed the impact of climate change, consumer demand, and policy regulations on the sustainable development of agri-food product supply chains. The analysis of barriers and challenges focused on limited resources, technological backwardness, and market competition, providing directions for policy-making and practical implementation. Analysis of driving factors revealed positive elements such as organizational models, quality assurance, and risk mitigation, serving as a basis for business’ strategic planning and policy formulation. Finally, the effect analysis examined the actual effects of sustainable supply chains on the environment, society, and the economy, offering insights for future policy-making and business development.

To promote the sustainability of the global agri-food supply chain and effectively apply the ABCDE framework, a comprehensive strategy and international cooperation are required. First and foremost, establishing a global knowledge platform guided by the Food and Agriculture Organization of the United Nations (FAO) is key to facilitating the exchange of knowledge and policy coordination. Such a platform can ensure that policies across nations are aligned with international standards, supporting the sustainability of the global agri-food supply chain. Secondly, technological innovation is central to enhancing the transparency and efficiency of the supply chain. The application of smart packaging technology and blockchain not only monitors environmental changes to products during logistics, but also provides a secure, transparent, and tamper-evident traceability method. This helps to build consumer trust and improve the quality and safety of agricultural products. Strengthening market regulation by standardizing market behavior through laws and regulations is crucial for ensuring the stability, efficiency, and safety of the supply chain. This includes improving the standardization and regulation of various aspects of the supply chain to ensure fair competition and environmental sustainability. Infrastructure construction is the cornerstone of modernizing the supply chain. Enhancing the infrastructure of the agri-food supply chain, such as cold chain logistics, storage facilities, and information platforms, can improve the operational efficiency and quality control of the supply chain. This is significant for reducing losses, extending shelf lives, and enhancing the market supply capacity of agricultural products. The reinforcement of the production and sales docking mechanism by establishing stable production and sales relationships and reducing intermediate links improves the market response speed and consumer satisfaction with agricultural products. This helps to increase the efficiency of the supply chain, reduce costs, and improve the market competitiveness of agricultural products. The application of green preservation technology can reduce the loss of fresh agricultural products, extend shelf lives, and ensure food safety and nutritional value. This not only helps to reduce waste but also increases the market supply capacity of agricultural products. Digital transformation is a trend in the development of supply chains. Utilizing information technology such as big data, cloud computing, and the Internet of things to achieve intelligent management and services in the supply chain can improve response speed and decision-making efficiency. Lastly, international cooperation is essential for jointly addressing global challenges. Through multilateral cooperation frameworks like the Belt and Road Initiative, strengthening exchanges and cooperation with countries around the world in the field of agri-food supply chains and sharing best practices can collectively enhance global agricultural productivity and food security. 

Considering the research structure above, future research directions in sustainable agricultural product supply chains are speculated to primarily involve the following aspects: strengthening international communication and collaboration to build a shared destiny for global food security and effectively respond to external shocks, as the global food supply chain urgently needs to enhance its resilience to ensure stability, prevent potential breakage, and enable rapid adjustment and recovery after being impacted. Strategically, it is possible to expand the agricultural product market and promote the upgrading and transformation of the agricultural industry. Given the perishable nature of food and the high standards of food safety, the post-harvest circulation process must adapt to the supply chain characteristics of specific regions [138]. Future research should focus on developing adaptable supply chain management strategies; integrating technological innovation to improve circulation efficiency, ensuring food quality and safety and promoting the sustainability and resilience of the global food supply chain; empowering the modern transformation of food production through digital means; implementing the concept of big food in the food industry chain, enhancing supervision and establishing a comprehensive assessment system; analyzing the excellent development mechanism factors of governments and businesses at international and regional levels, and investigating the demands of consumers and farmers to improve measures for stabilizing the food supply market and optimizing resource allocation, thereby achieving sustainable development goals and promoting social equity and economic prosperity.

### 6.2. Limitations and Outlook

This study provides crucial insights into the sustainability of the global agri-food supply chain; however, it has certain limitations. Firstly, the time range of the literature sources, focusing on the analysis of publications from January 2009 to July 2023, could be improved. Considering the scarcity and weak relevance of research literature before 2009, it should have been included in the analysis. Additionally, since the research cut-off date was July 2023, the understanding of publications after that year is relatively limited, imposing constraints on the accuracy and comprehensiveness of annual publication statistics. Future research plans involve expanding the study period, especially by comparing literature before 2009, to obtain a more scientifically informed trend analysis. Secondly, the limitations related to the types of literature should be acknowledged. Given the analytical nature of the CiteSpace software, this study primarily utilized journal articles from databases like SCI and SSCI as sample literature, mainly from English journals, with limited consideration for other types such as books, news articles, and magazines. This might have implications for the accuracy and comprehensiveness of the results. In future research, there are plans to integrate other software or fully utilize the updated features of CiteSpace to broaden the coverage of literature types for more comprehensive and accurate conclusions. Additionally, attention should be given to the limitations of the chosen database. This study solely relied on the resources of the Web of Science database and did not consider other well-known databases such as Scopus, Engineering, etc. This limitation affects the diversity of the content data acquired. In future research, there are plans to expand the data sources by incorporating more reputable databases, ensuring the broadness and authenticity of the data for a more comprehensive analysis of literature and content data. Finally, while the combination of CiteSpace and the ABCDE framework is powerful, it also has its limitations—CiteSpace may overlook the depth of qualitative analysis, and the qualitative framework might not fully leverage the broad data-driven perspective of CiteSpace, ultimately leading to an incomplete understanding of the research topic. The ABCDE framework has a degree of subjectivity and may require research to verify its feasibility. Future research should further integrate the strengths of both methods to overcome these limitations and provide a deeper understanding of the field of global agricultural sustainability.

## Figures and Tables

**Table 1:** Statistics of core authors from the top ten institutions.

Rank	Number of Publications (Articles)	The Year of First Publication	Author
1	7	2014	Accorsi, Riccardo
2	4	2016	Brunori, Gianluca
3	4	2016	Galli, Francesca
4	3	2021	Barrett, Christopher B.
5	3	2022	Ross, Helen
6	2	2014	De boer, I.J.M.
7	2	2016	Annaert, Bernd
8	2	2022	Baghizadeh, Komeyi
9	2	2022	Barba, Francisco J.
10	2	2019	Boerner, Jan

Data source: based on the WOS core database. Chart source: self-made by the author.

**Table 2:** Frequency and centrality chart of keywords.

Number	Count	Centrality	Year	Keywords	Number	Count	Centrality	Year	Keywords
1	119	0.13	2010	sustainability	31	20	0	2015	future
2	114	0.06	2014	management	32	19	0.02	2019	products
3	101	0.06	2010	life cycle assessment	33	18	0.03	2015	food systems
4	75	0.09	2011	supply chain	34	17	0.01	2021	environmental sustainability
5	66	0.03	2016	consumption	35	17	0.04	2015	behavior
6	62	0.08	2013	agriculture	36	17	0.07	2017	sustainable development
7	60	0.06	2010	food	37	16	0	2021	big data
8	57	0.06	2014	framework	38	16	0	2021	supply chain management
9	56	0.05	2014	systems	39	16	0.01	2018	technology
10	56	0.06	2012	food security	40	15	0.01	2022	lca
11	56	0.08	2014	impact	41	15	0.02	2015	drivers
12	54	0.02	2013	food waste	42	14	0.01	2021	networks
13	45	0.03	2012	climate change	43	14	0.03	2020	innovation
14	42	0.04	2010	food supply chain	44	13	0.01	2020	optimization
15	42	0.04	2012	quality	45	12	0	2019	assessment Ica
16	40	0.02	2015	challenges	46	12	0.01	2016	opportunity
17	36	0.03	2014	carbon footprint	47	12	0.01	2021	environmental impact
18	36	0.04	2010	greenhouse gas emissions	48	11	0.01	2019	indicators
19	35	0.03	2017	governance	49	11	0.02	2014	losses
20	34	0.04	2014	circular economy	50	10	0	2019	standards
21	33	0.02	2019	performance	51	10	0.01	2014	emissions
22	32	0.05	2018	waste	52	10	0.01	2015	carbon
23	29	0.02	2019	security	53	10	0.01	2018	policy
24	28	0.03	2019	environmental impacts	54	10	0.01	2019	land use
25	28	0.05	2012	supply chains	55	10	0.01	2019	biodiversity
26	27	0	2017	model	56	10	0.02	2013	ecosystem services
27	24	0.01	2018	system	57	10	0.03	2019	growth
28	24	0.03	2016	energy	58	10	0.04	2016	food system
29	24	0.06	2011	industry	59	9	0.01	2013	fish
30	22	0.01	2017	design	60	9	0.01	2020	knowledge

Data source: based on the WOS core database. Chart source: self-made by the author.

## Data Availability

No new data were created or analyzed in this study. Data sharing is not applicable to this article.
